# Clinical outcomes of preterm infants under 32 weeks who receive early enteral nutrition in a low- and middle-income country

**DOI:** 10.3389/fped.2026.1952558

**Published:** 2026-10-14

**Authors:** Javier Torres-Muñoz, Lorena Chamorro, Julián Torres-Figueroa, Julieth Ortega, Ruth Garzón, Paola Quintero

**Affiliations:** 1 INSIDE Group Research, School of Medicine, Faculty of Health, Universidad del Valle, Cali, Colombia; 2 Department of Pediatrics, School of Medicine, Faculty of Health, Universidad del Valle, Cali, Colombia; 3 Faculty of Health Sciences, Universidad ICESI, Cali, Colombia; 4 Versailles Clinic Cali, Cali, Colombia

**Keywords:** enteral feeding, growth, low and mid income countries, newborn, nutrition, preterm

## Abstract

**Introduction:**

Early initiation of enteral nutrition in preterm infants has been considered as a risk factor due to its possible association with adverse outcomes.

**Objective:**

To determine the clinical outcomes in preterm infants younger than 32 weeks and/or with a birth weight under 1,500 g who received enteral nutrition within the first 48 h in two neonatal units in a middle-income country.

**Materials and methods:**

This retrospective two-center cohort study compared clinical outcomes between preterm infants with a gestational age ≤32 weeks and/or birth weight <1,500 g who initiated enteral feeding within or after 48 h of life. Categorical variables were compared using the chi-square or Fisher's exact test, and continuous variables using the Mann–Whitney U test. As the primary analysis, separate multivariable logistic regression models with robust standard errors were fitted for NEC, sepsis, BPD, and mortality. All models were adjusted for gestational age, birth weight, 5 min Apgar score, and study institution; the sepsis and BPD models were additionally adjusted for neonatal resuscitation. Propensity scores were estimated among 159 infants with complete data using prespecified baseline characteristics and study institution. One-to-one nearest-neighbor matching with replacement was performed using a caliper of 0.05 and restriction to common support. Covariate balance was assessed using standardized mean differences, and matched outcomes were analyzed using matching weights and robust standard errors. Associations were reported as odds ratios with 95% confidence intervals.

**Results:**

Among 173 preterm infants, 117 (67.6%) initiated enteral feeding within 48 h of life and 56 (32.4%) initiated it thereafter. In the primary full-cohort multivariable analyses, delayed initiation was not significantly associated with necrotizing enterocolitis (aOR 1.13; 95% CI, 0.32–4.02; *p* = 0.848), sepsis (aOR 1.32; 95% CI, 0.54–3.26; *p* = 0.542), bronchopulmonary dysplasia (aOR 1.08; 95% CI, 0.42–2.79; *p* = 0.878), or mortality (aOR 0.68; 95% CI, 0.21–2.24; *p* = 0.523). The propensity score–matched sensitivity analysis included 38 infants with delayed feeding initiation and 38 weighted control observations derived from 18 unique infants with early feeding initiation. In this analysis, delayed initiation was associated with a longer time to achieve full enteral nutrition (weighted mean difference, 6.19 days; 95% CI, 0.10–12.27; *p* = 0.046) and higher odds of bronchopulmonary dysplasia (OR 8.25; 95% CI, 1.54–44.29; *p* = 0.014).

**Conclusions:**

Delayed enteral feeding was not associated with major neonatal outcomes in the primary analyses. Sensitivity findings suggesting delayed achievement of full enteral nutrition and increased BPD require cautious interpretation

## Introduction

Preterm newborns with very low birth weight (VLBW) experience significant nutritional deficits during the first weeks of life, posing a relevant clinical challenge. This situation highlights the need to implement early nutrition strategies that promote adequate postnatal growth and reduce the risk of associated complications. Several observational studies have shown that critically ill preterm newborns with VLBW rarely achieve adequate nutritional intake during the initial phases of postnatal life (1).

The current evidence indicates that cumulative deficits in energy and protein intake, observed shortly after birth, account for approximately 50% of the decline in weight-for-age from birth to hospital discharge (2). These deficiencies could contribute not only to suboptimal postnatal growth but also to an increased risk of adverse clinical outcomes (1).

From a physiological perspective, nutrition in preterm neonates with VLBW should aim to replicate, as closely as possible, the intrauterine growth pattern. Preterm birth disrupts fetal development during the third trimester, a critical stage for the accelerated maturation of organs and systems. Consequently, adequate nutritional support is essential to improve short- and long-term outcomes and reduce morbidity and mortality in this highly vulnerable population (3).

Currently, there is consensus on the need to initiate enteral nutrition as early as possible, given its multiple benefits beyond caloric and protein intake. Reported advantages include the trophic stimulation of the gastrointestinal tract, the promotion of a healthy gut microbiota, the strengthening of the mother-infant bond, the development of physiological digestive rhythms, the acquisition of feeding habits, and the maturation of neurological functions associated with sucking and swallowing (3).

Clinical trials conducted in low- and middle-income countries have shown that the early and exclusive initiation of enteral nutrition may be associated with a reduction in the length of hospitalization and greater weight gain, without an associated increase in the risk of necrotizing enterocolitis (NEC) (4). In addition, various publications have highlighted the importance of having institutional enteral nutrition protocols that standardize clinical practice and help minimize complications such as NEC, sepsis, and the unnecessary prolongation of hospital stay (5–9).

The main objective of the study is to evaluate the effect of early initiation of enteral feeding—within the first 48 h of life in preterm neonates under 32 weeks of gestation or with very low birth weight, in the context of a low- and middle-income country.

## Methodology

The study was conducted with the approval of the Ethics Committee of the Hospital Universitario del Valle (HUV) and Clínica Versalles and was carried out in the neonatal intensive care unit (CIRENA) of the Hospital Universitario del Valle, and the intensive care unit of Clínica Versalles (UCIREN). These institutions report up to 60% of the city's births. The study design is an analytical observational cohort of preterm newborns under 32 weeks of gestational age or under 1,500 g of birth weight, hospitalized in neonatal ICUs between April 1, 2023, to December 31, 2024 who received enteral feeding before or after 48 h—either maternal milk, donated milk, or formula—and whose information was collected from medical records and institutional databases.

Feeding protocols by institution. At Hospital Universitario del Valle, enteral feeding was initiated according to clinical criteria, using the mother's own milk/preterm formula/donor human milk, preferably using the mother's own milk. Feedings were started at approximately 20–30 mL/kg/day and advanced by 30 mL/kg/day, according to clinical tolerance. At Clínica Versalles, enteral feeding was initiated according to clinical criteria, using the mother's own milk/preterm formula/no human milk bank available, with an initial volume of 30 mL/kg/day and daily increments of 30 mL/kg/day. In both institutions, the decision to delay, interrupt, or advance feeding was made by the attending neonatal team based on the infant's hemodynamic stability and gastrointestinal tolerance.

Inclusion criteria:

- Patients with a gestational age less than or equal to 32 weeks, determined by amenorrhea, first-trimester ultrasound, or Ballard score.
- Patients with a birth weight less than or equal to 1,500 g, regardless of gestational age.
- Patients who are hospitalized in the neonatal units of Clínica Versalles or the Hospital Universitario del Valle.

Exclusion criteria:

- Patients with major congenital malformations.
- Patients with metabolic diseases.
- Newborns who died within the first 48 h of life.

### Target population of the study

Preterm newborns with a gestational age less than or equal to 32 weeks, hospitalized in neonatal ICUs and receive breast milk, donor human milk, or formula before or after 48 h. Patients with major congenital malformations or metabolic diseases were excluded from the study.

### Sample size

All newborns who met the selection criteria at the two institutions during the study period were consecutively included. No probabilistic sampling was performed. Although a target sample of approximately 180 participants was estimated during planning, subsequent review identified that the proportions used did not correspond to NEC incidences under the 48 h cutoff employed in this study. Therefore, these proportions were not used to interpret the specific power of the NEC analysis.

### Statistical analysis

An exploratory analysis was conducted to assess the distributions of continuous variables, frequency distributions of categorical variables, missing data, and data consistency. Categorical variables were summarized as frequencies and percentages and compared using the chi-square or Fisher's exact test, as appropriate. Continuous variables were summarized as medians and interquartile ranges and compared using the Mann–Whitney *U*-test.

As the primary analysis, separate outcome-specific multivariable logistic regression models were fitted in the full cohort to evaluate the association between delayed enteral feeding initiation (>48 h) and necrotizing enterocolitis (NEC), sepsis, bronchopulmonary dysplasia (BPD), and mortality. All models included feeding initiation group, gestational age, birth weight per 100 g increase, 5 min Apgar score, and study institution. The sepsis and BPD models additionally included neonatal resuscitation. Neonatal resuscitation was excluded from the NEC and mortality models because it perfectly predicted these outcomes. Conventional logistic regression with robust standard errors was used, and results were reported as adjusted odds ratios (aORs) with 95% confidence intervals. Early enteral feeding initiation (≤48 h) and Clínica Versalles were used as the reference categories. Complete-case analysis was performed separately for each model; therefore, the number of observations varied across outcomes.

As a sensitivity analysis to reduce confounding by indication related to baseline clinical severity, propensity scores for delayed enteral feeding initiation (>48 h) were estimated among 159 infants with complete data using logistic regression. Prespecified covariates included gestational age, birth weight, 5 min Apgar score, neonatal resuscitation, respiratory distress syndrome, noninvasive ventilation from birth, antenatal corticosteroid exposure, prenatal care, and study institution. One-to-one nearest-neighbor matching with replacement was performed using a caliper of 0.05 on the estimated propensity score and restricting the analysis to the region of common support. This approach estimated the average treatment effect on the treated (ATT), with infants with delayed feeding initiation considered the treated group. Covariate balance was assessed using standardized mean differences and Rubin's B and R statistics. Infants with delayed feeding initiation received a weight of 1, whereas early-fed controls were weighted according to the number of times they were selected as matches. Continuous and dichotomous outcomes were analyzed using weighted linear and logistic regression, respectively, with robust standard errors. Propensity score overlap was assessed graphically using kernel density plots before and after matching, applying matching weights to the post-matching distribution. All analyses were performed using Stata/SE 14.0 (StataCorp LP, College Station, TX, EE. UU.).

## Results

Of the 180 newborns initially identified, 173 had available information on the timing of enteral feeding initiation and were included in the primary analysis: 117 (67.6%) initiated enteral feeding within 48 h of birth and 56 (32.4%) initiated it after 48 h. Information on the birth institution was unavailable for one newborn; therefore, analyses that included this variable comprised a maximum of 172 participants. Among the 159 preterm infants with complete data for all variables included in the propensity score model, one-to-one nearest-neighbor matching with replacement matched 38 infants with delayed feeding initiation to control observations drawn from 18 unique infants with early feeding initiation. Thus, the matched sample comprised 56 unique infants. Because some early-fed controls were selected more than once, their matching weights summed to 38 control observations in the weighted analysis (Figure 1).

**Figure 1:**
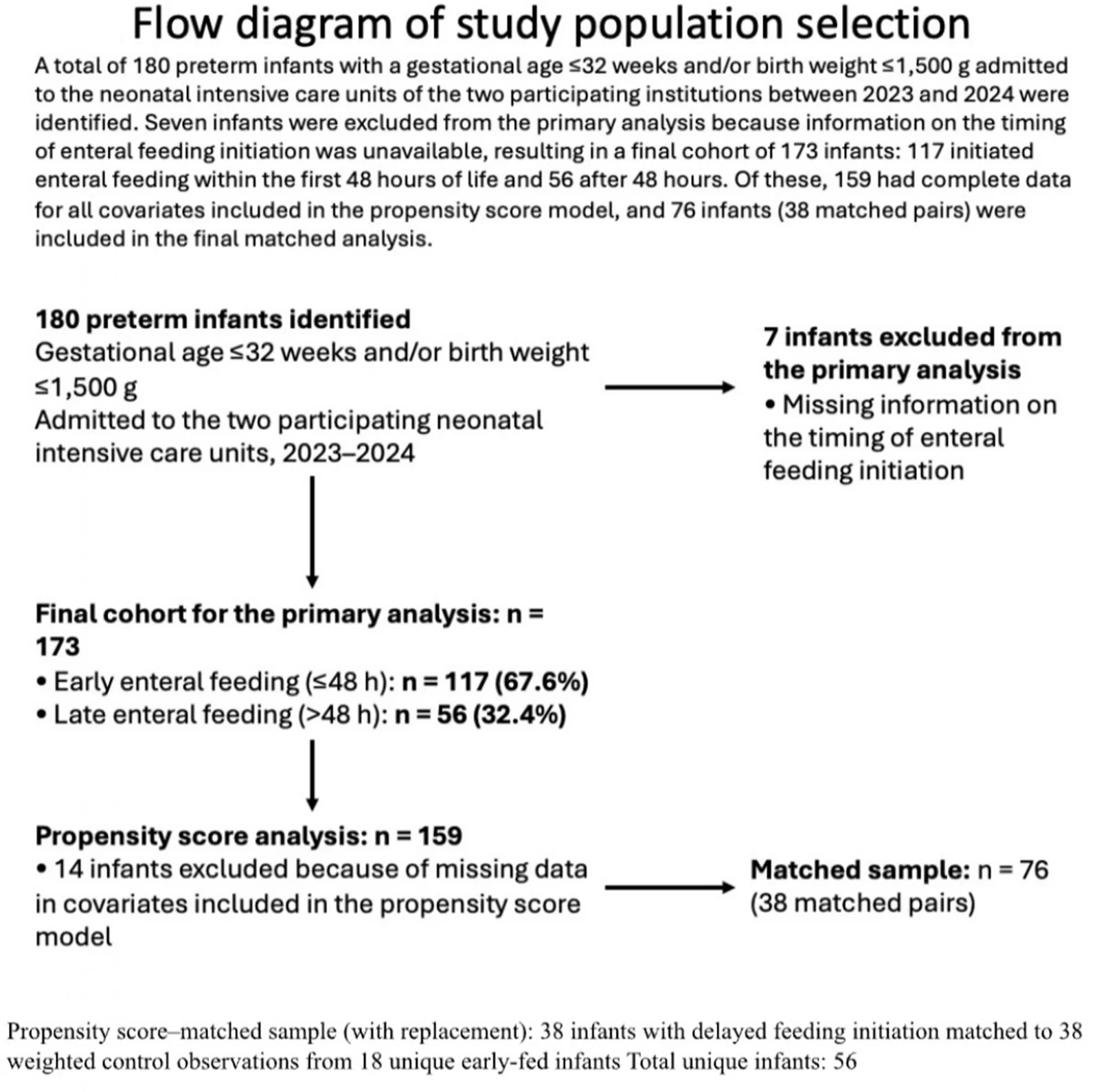
Flow diagram of study population selection. A total of 180 preterm infants with a gestational age ≤32 weeks and/or birth weight ≤1,500 g admitted to the neonatal intensive care units of the two participating institutions between 2023 and 2024 were identified. Seven infants were excluded from the primary analysis because information on the timing of enteral feeding initiation was unavailable, resulting in a final cohort of 173 infants: 117 initiated enteral feeding within the first 48 h of life and 56 after 48 h. Of these, 159 had complete data for all covariates included in the propensity score model, and 76 infants (38 matched pairs) were included in the final matched analysis. Propensity score-matched sample (with replacement): 38 infants with delayed feeding initiation matched to 38 weighted control observations from 18 unique early-fed infants Total unique infants: 56.

Perinatal characteristics (Table 1) were generally similar between the two groups, with no significant differences in gestational age, birth weight, 5 min Apgar score, prenatal care, antenatal corticosteroid exposure, or requirement for noninvasive ventilation from birth. However, newborns who initiated enteral feeding after 48 h were more frequently born at Hospital Universitario del Valle (91.1% vs. 38.8%; *p* < 0.001), resided outside Cali (46.2% vs. 30.2%; *p* = 0.049), and required neonatal resuscitation more frequently (92.9% vs. 60.9%; *p* < 0.001).

**Table 1:** Comparison of perinatal and clinical characteristics according to the timing of initiation of enteral feeding.

Variable	≤48 h (*n* = 117)	>48 h (*n* = 56)	*P* value
Perinatal characteristics			
Gestational age, weeks, median (IQR)	28 (27–29)	28 (27–30)	0.818
Gestational age <28 weeks, n/N (%)	47/117 (40.2)	23/56 (41.1)	0.910
Birth weight, g, median (IQR)	1,115 (884–1,290)	1,072.5 (777.5–1,200)	0.149
Birth weight <1,000 g, n/N (%)	40/117 (34.2)	20/56 (35.7)	0.844
5 min Apgar score, median (IQR)	8 (7–9), *n* = 116	8 (7–9), *n* = 52	0.867
Poor prenatal care (≤3 visits), n/N (%) 28/111 (25.2)		19/56 (33.9)	0.230
No antenatal corticosteroid exposure, n/N (%)	27/116 (23.3)	14/56 (25.0)	0.804
Residence outside Cali, n/N (%)	32/106 (30.2)	24/52 (46.2)	0.049
Birth institution, n/N (%)			<0.001
Clínica Versalles	71/116 (61.2)	5/56 (8.9)	
Hospital Universitario del Valle	45/116 (38.8)	51/56 (91.1)	
Neonatal resuscitation, n/N (%)	70/115 (60.9)	52/56 (92.9)	<0.001
Noninvasive ventilation required from 82/117 (70.1) birth, n/N (%)	40/56 (71.4)	0.856	
Neonatal clinical variables			
Respiratory distress syndrome, (%)	n/N 72/117 (61.5)	48/56 (85.7)	0.001
Bronchopulmonary dysplasia, (%)	n/N 25/114 (21.9)	22/54 (40.7)	0.011
Necrotizing enterocolitis, n/N (%)	7/117 (6.0)	9/56 (16.1)	0.047
Intraventricular hemorrhage, (%)	n/N 13/117 (11.1)	12/56 (21.4)	0.071
Sepsis, n/N (%)	22/116 (19.0)	23/55 (41.8)	0.002
Hemodynamically significant patent 27/108 (25.0) ductus arteriosus, n/N (%)	15/54 (27.8)	0.704	
Retinopathy of prematurity, n/N (%) 10/117 (8.5	3/56 (5.4)	0.552	
Interventions and outcomes			
Endotracheal intubation >7 days, n/N 37/100 (37.0) (%)	17/43 (39.5)	0.774	
Hospital stay >15 days, n/N (%) 106/116 (91.4)	46/55 (83.6)	0.132	
Mortality, n/N (%)	15/115 (13.0)	11/55 (20.0)	0.238
Human milk, n/N (%)	16/110 (14.5)	14/54 (25.9)	0.076

Values are presented as median (interquartile range [IQR]) for continuous variables and n/N (%) for categorical variables. Percentages were calculated using the number of infants with available data within each enteral-feeding group as the denominator. Continuous variables were compared using the Mann–Whitney *U*-test. Categorical variables were compared using Pearson's chi-square test or Fisher's exact test, as appropriate. Denominators vary because of missing data.

a Fisher's exact test.

A *P* value < 0.05 was considered statistically significant.

In the unadjusted analysis, the group with enteral feeding initiated after 48 h had higher proportions of respiratory distress syndrome (85.7% vs. 61.5%; *p* = 0.001), bronchopulmonary dysplasia (40.7% vs. 21.9%; *p* = 0.011), necrotizing enterocolitis (16.1% vs. 6.0%; *p* = 0.047), and sepsis (41.8% vs. 19.0%; *p* = 0.002). No statistically significant differences were observed in the remaining clinical interventions and outcomes, including mortality (20.0% vs. 13.0%; *p* = 0.238).

The propensity score model included gestational age, birth weight, 5 min Apgar score, neonatal resuscitation, respiratory distress syndrome, noninvasive ventilation from birth, antenatal corticosteroid exposure, prenatal care, and study institution. One-to-one nearest-neighbor matching with replacement was performed using a caliper of 0.05 and restriction to the region of common support. The procedure matched 38 infants with delayed feeding initiation to control observations drawn from 18 unique infants with early feeding initiation. Thus, the matched sample comprised 56 unique infants. Because matching was performed with replacement, some early-fed controls were selected more than once, resulting in a weighted control count of 38. After matching, all absolute standardized mean differences were below 10%, indicating adequate covariate balance (Table 2).

**Table 2:** Covariate balance before and after propensity score matching .

Covariate	Before matching: delayed	Before matching: early	SMD, %	After matching: delayed	After matching: early^a^	SMD, %
Gestational age, weeks, mean	28.30	28.20	4.0	29.09	28.86	9.9
Birth weight, g, mean	1,022.1	1,091.9	−26.1	1,135.7	1,122.9	4.8
5 min Apgar score, mean	7.56	7.81	−14.4	7.87	7.82	3.0
Neonatal resuscitation, proportion	0.942	0.636	80.7	0.921	0.921	0.0
Respiratory distress syndrome, proportion	0.904	0.626	68.9	0.868	0.895	−6.5
Noninvasive ventilation from birth, proportion	0.712	0.692	4.3	0.605	0.579	5.7
Absence of antenatal corticosteroids, proportion	0.250	0.252	−0.5	0.316	0.316	0.0
Poor prenatal care proportion	0.365	0.365	24.5	0.289	0.263	5.7
Hospital Universitario del Valle, proportion	0.962	0.421	143.6	0.947	0.947	0.0

a Propensity score matching with replacement included 38 infants with delayed feeding initiation and 18 unique infants with early feeding initiation. Controls selected more than once were weighted according to the number of times they were selected, resulting in a weighted control count of 38 observations.

Propensity score distributions showed limited overlap before matching but close alignment between delayed-fed infants and weighted early-fed controls after matching (Figure 2).

**Figure 2:**
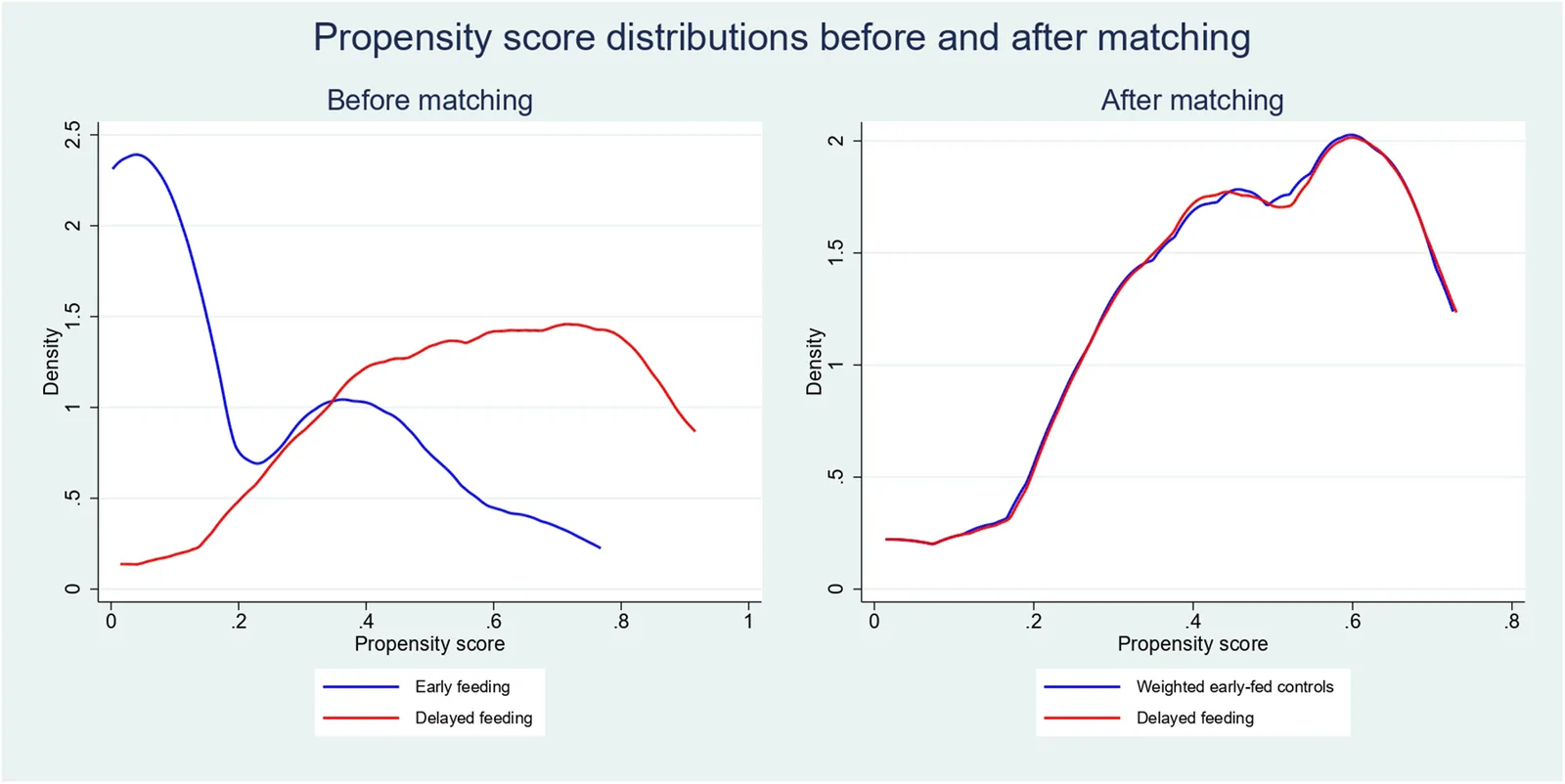


After matching, the mean standardized bias decreased from 40.8% to 4.0%, with no overall imbalance between groups (*p* = 1.000). All post-matching standardized mean differences were below 10%. The SMD for neonatal resuscitation decreased from 80.7% to 0%, that for respiratory distress syndrome from 68.9% to −6.5%, and that for institution of birth from 143.6% to 0%.

In the propensity score–matched sensitivity analysis (Table 3), delayed enteral feeding initiation was associated with a longer time to achieve full enteral nutrition (weighted means, 19.76 vs. 13.57 days; mean difference, 6.19 days; 95% CI: 0.10–12.27; *p* = 0.046)) and higher odds of BPD (weighted proportions, 33.3% vs. 5.7%; OR 8.25; 95% CI: 1.54–44.29; *p* = 0.014). Mortality was lower in the delayed-initiation group (13.2% vs. 42.1%; OR 0.21; 95% CI: 0.05–0.93; *p* = 0.040). No significant differences were observed in hospital length of stay (weighted means, 45.00 vs. 32.08 days; *p* = 0.064), NEC (*p* = 0.558), or sepsis (*p* = 0.270).

**Table 3:** Clinical outcomes in the propensity score–matched sensitivity analysis.

Outcome	Early initiation ≤48 h, weighted estimate	Late initiation >48 h, weighted estimate	Weighted effect estimate (95% CI)	*p* value	Unique *n*
Time to full enteral nutrition, weighted mean, days	13.57	19.76	MD 6.19 (0.10–12.27)	0.046	47
Length of hospital stay, weighted mean, days	32.08	45.00	MD 12.92 (−0.76 to 26.60)	0.064	56
Necrotizing enterocolitis, weighted %	23.7	15.8	OR 0.60 (0.11–3.26)	0.558	56
Sepsis, weighted %	21.1	39.5	OR 2.45 (0.50–11.98)	0.270	56
Bronchopulmonary dysplasia, weighted %	5.7	33.3	OR 8.25 (1.54–44.29)	0.014	53
Mortality, weighted %	42.1	13.2	OR 0.21 (0.05–0.93)	0.040	56

MD: weighted mean difference, calculated as late minus early initiation.

Data is presented as weighted means or percentages. Matching with replacement included 38 infants with delayed feeding initiation and 18 unique early-fed controls. Outcomes were analyzed using matching-weighted linear or logistic regression with robust standard errors and are reported as mean differences (MDs) or odds ratios (ORs), respectively. Sample sizes varied because of missing data. Mortality was considered exploratory because survival bias related to the 48 h exposure-classification period could not be excluded. CI, confidence interval.

Delayed initiation of enteral feeding differed substantially between institutions, occurring in 53.1% of newborns at Hospital Universitario del Valle and 6.6% of those at Clínica Versalles (*p* < 0.001). In the outcome-specific multivariable logistic regression analyses (Table 4), delayed initiation of enteral feeding was not significantly associated with NEC (aOR 1.13; 95% CI: 0.32–4.02; *p* = 0.848), sepsis (aOR 1.32; 95% CI: 0.54–3.26; *p* = 0.542), BPD (aOR 1.08; 95% CI: 0.42–2.79; *p* = 0.878), or mortality (aOR 0.68; 95% CI: 0.21–2.24; *p* = 0.523).

**Table 4:** Outcome-specific multivariable logistic regression analyses.

Variable	NEC, *n* = 167 aOR (95% CI); p	Sepsis, *n* = 166 aOR (95% CI); p	BPD, *n* = 161 aOR (95% CI); p	Mortality, *n* = 166 aOR (95% CI); p
Delayed enteral feeding initiation (>48 h)	1.13 (0.32–4.02); 0.848	1.32 (0.54–3.26); 0.542	1.08 (0.42–2.79); 0.878	0.68 (0.21–2.24); 0.523
Gestational age, per week	1.01 (0.71–1.43); 0.950	0.88 (0.70–1.12); 0.294	0.74 (0.57–0.97); 0.026	0.80 (0.57–1.12); 0.197
Birth weight, per 100 g	0.74 (0.56–0.98); 0.033	0.87 (0.71–1.07); 0.177	0.83 (0.67–1.03); 0.095	0.66 (0.49–0.90); 0.008
5 min Apgar score	1.10 (0.78–1.55); 0.589	0.88 (0.67–1.16); 0.378	1.04 (0.79–1.38); 0.779	0.96 (0.74–1.24); 0.749
Neonatal resuscitation	—	1.51 (0.38–5.97); 0.555	1.23 (0.41–3.70); 0.712	—
Hospital Universitario del Valle	7.73 (1.35–44.45); 0.022	6.38 (1.94–21.01); 0.002	5.29 (1.69–16.60); 0.004	4.78 (1.49–15.38); 0.009

Values are adjusted odds ratios (aORs), 95% confidence intervals, and *p* values. Separate conventional logistic regression models with robust standard errors were fitted for each outcome. Early enteral feeding initiation (≤48 h) and Clínica Versalles were the reference categories. All models included gestational age, birth weight per 100 g increase, 5 min Apgar score, and study institution. The sepsis and BPD models additionally included neonatal resuscitation. Neonatal resuscitation was excluded from the NEC and mortality models because of perfect prediction. NEC, necrotizing enterocolitis; BPD, bronchopulmonary dysplasia.

Higher birth weight was associated with lower odds of NEC (aOR per 100-g increase, 0.74; 95% CI: 0.56–0.98; *p* = 0.033) and mortality (aOR 0.66; 95% CI: 0.49–0.90; *p* = 0.008), whereas greater gestational age was associated with lower odds of BPD (aOR per week, 0.74; 95% CI: 0.57–0.97; *p* = 0.026). Birth at Hospital Universitario del Valle was associated with higher odds of NEC (aOR 7.73; 95% CI: 1.35–44.45; *p* = 0.022), sepsis (aOR 6.38; 95% CI: 1.94–21.01; *p* = 0.002), BPD (aOR 5.29; 95% CI: 1.69–16.60; *p* = 0.004), and mortality (aOR 4.78; 95% CI: 1.49–15.38; *p* = 0.009).

## Discussion

Enteral nutrition is the ideal way to provide nutrients to preterm infants, especially those with very low birth weight. Early initiation and advancement of enteral feeding have an impact on the health of the preterm infant by improving nutrient intake, which affects their growth and neurodevelopment.

The objective of this study was to identify clinical outcomes related to early enteral feeding (before 48 h of life) in preterm newborns under 32 weeks of gestational age and weighing less than 1,500 g.

This study, conducted in a middle-income country, found that initiating enteral feeding within the first 48 h of life was associated with earlier achievement of full enteral nutrition, without an increased frequency of major neonatal complications compared with later initiation. Publications from studies carried out in low- and middle-income countries have identified that early initiation—and in some cases exclusive enteral feeding—results in better clinical outcomes (10–12). Unlike feeding protocols for preterm infants born in some high-income countries, which initiate parenteral nutrition shortly after birth and introduce enteral nutrition gradually (10–13). In our study, NEC was more frequent among infants with delayed enteral feeding initiation in the unadjusted analysis; however, this association was no longer statistically significant in the propensity score-matched sensitivity analysis (weighted OR 0.60; 95% CI 0.11–3.26; *p* = 0.558). Therefore, our findings do not support an independent association between feeding-initiation timing and NEC. However, because the precise timing of NEC onset was unavailable, reverse causality cannot be excluded. This result is consistent with previous studies suggesting that early enteral feeding is not associated with an increased risk of NEC (14, 15).

In a publication of six clinical trials conducted in India (4) They found that early and exclusive enteral nutrition did not lead to an increased risk of developing NEC. In 2022, a systematic review with meta-analysis was published, aiming to evaluate the effect of delayed introduction of progressive enteral feeding (defined as >4 days of life) on the risk of necrotizing enterocolitis (NEC), mortality, and other morbidities in very preterm or very low birth weight (VLBW) newborns. The analysis included 14 randomized clinical trials with a total of 1,551 neonates. The results showed that delaying the introduction of progressive enteral feeding was not associated with a significant reduction in the risk of NEC (relative risk [RR]: 0.81; 95% confidence interval [CI]: 0.58–1.14). The authors concluded that delaying the initiation of progressive enteral feeding beyond four days of life does not reduce the risk of NEC in this population (16).

As evidenced in the present study, early progression of enteral feeding to 20–30 mL/kg/day before 48 h of life was not associated with adverse outcomes such as necrotizing enterocolitis (NEC). These findings are consistent with those reported in a meta-analysis that included six randomized clinical trials with a total of 526 newborns (16). In those trials, the progression rate was comparable to that of our study, ranging between 20 and 30 mL/kg/day, and in some cases reaching up to 60–80 mL/kg/day, initiated shortly after birth using breast milk, donor milk, or formula.

Although sepsis was more frequent among newborns who initiated enteral feeding after 48 h in the unadjusted analysis, this difference was no longer statistically significant after propensity score matching. In contrast, a 2022 meta-analysis by Walsh et al. (4), which included seven randomized clinical trials and 872 very preterm or very low birth weight newborns, found that delaying enteral feeding beyond four days increased the risk of invasive infection (OR 1.44; 95% CI: 1.15–1.80). However, the different definitions of delayed feeding limit direct comparison with our findings.

In a meta-analysis published by Ramaa Chitale et al. in 2022 (17), 14 randomized controlled clinical trials with 1,505 participants were included. They compared early initiation, defined by the authors as before 72 h, vs. late initiation, defined as after 72 h. They found that early initiation reduced sepsis, with a significant reduction in mortality (RR 0.69; 95% CI: 0.48–0.99).

In the propensity score-matched sensitivity analysis, delayed feeding initiation was associated with higher odds of bronchopulmonary dysplasia (BPD), defined as the need for supplemental oxygen at 36 weeks' postmenstrual age a (OR 8.25; 95% CI 1.54–44.29; *p* = 0.014), The association with bronchopulmonary dysplasia observed only in the matched sensitivity analysis was imprecise and should be interpreted cautiously because of the small number of unique controls, limited propensity-score overlap, and possible residual confounding. An observational study (18) of 1,366 newborns evaluated differences in nutritional support during the first week of life according to clinical severity. After adjustment for critical illness, the risk of BPD and other morbidities decreased by approximately 2% with each increase in total energy intake. Although these findings suggest a potential benefit of adequate early nutritional support, the study evaluated overall energy intake rather than the timing of enteral feeding initiation; therefore, its results are not directly comparable with ours. In 2015, Konnikova et al. (19) conducted an observational study involving 130 infants born before 33 weeks of gestation. Initiation of enteral feeding after the third postnatal day was associated with a 4.5-fold increase in chronic lung disease and other morbidities compared with initiation before the third postnatal day (OR 4.5; 95% CI: 1.8–11.5; *p* = 0.002).

In our cohort, RDS was more frequent among newborns who initiated enteral feeding after 48 h in the unadjusted analysis. However, because RDS generally preceded the decision to initiate enteral feeding, it should be considered a potential determinant of delayed feeding rather than a consequence of it. This finding may reflect greater caution among healthcare teams when initiating enteral feeding in newborns with respiratory compromise, as well as confounding by clinical severity. After propensity score matching, RDS was adequately balanced between the groups.

Several studies have emphasized the importance of appropriate early nutrition in premature neonates, primarily with maternal or donor milk, highlighting its impact on brain development, cognitive function, and future neurodevelopment (9, 19–21).

The lower mortality observed in the delayed-initiation group should be considered exploratory because of the small effective control sample and the possibility of residual confounding and survival bias.

Differences between institutions in feeding initiation and neonatal outcomes may reflect variations in patient complexity, referral patterns, and clinical protocols rather than a direct institutional effect. Despite adjustment for study center, residual confounding, limited overlap, and wide confidence intervals warrant cautious interpretation of these associations.

Among the limitations of the study is its observational design, which may favor the presence of bias and is affected by the availability of incomplete medical records. This limitation prevented the collection of sufficient information for several of the variables analyzed and may have influenced the results. That adjustment for the available category produced similar results, but generalizability to centers with high use of human milk remains limited. The retrospective database did not provide precise onset times for all clinical complications. Consequently, temporal precedence could not be established for every episode of sepsis or necrotizing enterocolitis, and reverse causality remains possible because early clinical deterioration may have delayed the initiation of enteral feeding.

The small number of events, particularly for NEC, limited statistical power and produced imprecise estimates. Therefore, nonsignificant findings should not be interpreted as evidence of equivalence or absence of association.

Among the strengths of this study, its findings provide context-specific evidence that may inform the standardization of enteral feeding protocols for preterm and low-birth-weight infants, particularly in low- and middle-income settings. However, given the observational design, these findings should not be interpreted as definitive evidence of safety or clinical benefit.

## Conclusions

In this cohort of preterm infants born at ≤32 weeks of gestation and/or with a birth weight ≤1,500 g, delayed enteral feeding initiation was not independently associated with necrotizing enterocolitis, sepsis, or bronchopulmonary dysplasia in the primary full-cohort analyses adjusted for baseline characteristics and study institution. In the propensity score–matched sensitivity analysis, delayed initiation was associated with a longer observed time to achieve full enteral nutrition. An imprecise association between delayed initiation and bronchopulmonary dysplasia was observed in the matched analysis but was not confirmed in the primary analysis.

Marked differences in feeding-initiation practices were observed between the participating institutions, emphasizing the importance of institutional protocols and baseline clinical severity. Overall, the findings do not provide consistent evidence that enteral feeding initiation within the first 48 h is associated with an increased frequency of the evaluated complications. However, they should not be interpreted as demonstrating safety or causal benefit because of the retrospective design, limited propensity-score overlap, small number of unique matched controls, center-level differences, potential reverse causality, and residual confounding. Larger prospective multicenter studies in low- and middle-income settings are needed to confirm these findings and inform standardized feeding protocols.

## Data Availability

The original contributions presented in the study are included in the article/Supplementary Material, further inquiries can be directed to the corresponding author/s.
